# Comparative analysis of barium titanate thin films dry etching using inductively coupled plasmas by different fluorine-based mixture gas

**DOI:** 10.1186/1556-276X-9-530

**Published:** 2014-09-26

**Authors:** Yang Li, Cong Wang, Zhao Yao, Hong-Ki Kim, Nam-Young Kim

**Affiliations:** 1 Department of Electronic Engineering, Kwangwoon University, 20 Gwangun-Ro, Nowon-gu, Seoul 139-701, Republic of Korea; 2 Department of Electronic Materials Engineering, Kwangwoon University, 20 Gwangun-Ro, Nowon-gu, Seoul 139-701, Republic of Korea

**Keywords:** Barium titanate, Fluorine-based mixture gas, Inductively coupled plasma etching

## Abstract

In this work, the inductively coupled plasma etching technique was applied to etch the barium titanate thin film. A comparative study of etch characteristics of the barium titanate thin film has been investigated in fluorine-based (CF_4_/O_2_, C_4_F_8_/O_2_ and SF_6_/O_2_) plasmas. The etch rates were measured using focused ion beam in order to ensure the accuracy of measurement. The surface morphology of etched barium titanate thin film was characterized by atomic force microscope. The chemical state of the etched surfaces was investigated by X-ray photoelectron spectroscopy. According to the experimental result, we monitored that a higher barium titanate thin film etch rate was achieved with SF_6_/O_2_ due to minimum amount of necessary ion energy and its higher volatility of etching byproducts as compared with CF_4_/O_2_ and C_4_F_8_/O_2_. Low-volatile C-F compound etching byproducts from C_4_F_8_/O_2_ were observed on the etched surface and resulted in the reduction of etch rate. As a result, the barium titanate films can be effectively etched by the plasma with the composition of SF_6_/O_2_, which has an etch rate of over than 46.7 nm/min at RF power/inductively coupled plasma (ICP) power of 150/1,000 W under gas pressure of 7.5 mTorr with a better surface morphology.

## Background

Recently, gate insulator materials of downscaling MOSFET devices and insulator materials for metal-insulator-metal (MIM) capacitor have become key issues in semiconductor memory application field. The existence of gate dielectric suffers from increased gate leakage [1], and the insulator of MIM also cannot meet the requirement of high capacitance density and low leakage current [2-4]. To solve these challenges, high-k materials are needed for gate insulator and insulator of MIM capacitor. Until now, high-k materials including TiO_2_, TiN, HfAlO_3_, BaSeTiO_3_ and BaTiO_3_ have been widely studied [5-9]. Among these materials, BaTiO_3_ is emerging as a promising material due to the merits of high dielectric constant, low leakage current and excellent piezoelectric and ferroelectric properties [10-12]. Using BaTiO_3_ thin film as the gate insulator and insulator of MIM capacitor can greatly improve the performance and the density of integrated circuit. So far, although a great deal of researchers devoted to researching the characteristics of BaTiO_3_ thin film for using different applications, there has been little study on micropatterning properties of BaTiO_3_. A research presents an investigation of the chemical mechanical polishing (CMP) process [13]. However, this CMP method has a significant limitation and complicated fabrication process. With regard to the etching technology, only in [14], a study on characterization of dry etching process is presented, but the authors just give a simple presentation about the relationship between plasma etch rate and applied RF power and mixture gas mixing ratio; there is no deep and systematic characterization for etching mechanism. To date, there is no feasible technology known for the etching of BaTiO_3_ thin film. These obstacles hinder understanding the properties of the BaTiO_3_ thin film etching process and further impede the related optimization of process. Therefore, it is necessary to study on how obtain a high etch rate and a good etch profile for dry etching mechanism of BaTiO_3_ thin film.

In this research, BaTiO_3_ thin films were etched using inductively coupled plasma (ICP) system with different fluorine-based plasmas. The etch rates of BaTiO_3_ thin films etched in different fluorine-based (CF_4_/O_2_, C_4_F_8_/O_2_ and SF_6_/O_2_) plasmas were compared. A comparative study of etch characteristics of the BaTiO_3_ thin films in these plasmas was conducted. The surface morphology of BaTiO_3_ thin films was examined by atomic force microscopy (AFM). Also, the chemical compositions and the binding states of the corresponding elements on the surface for each etched films were analysed by X-ray photoelectron spectroscopy (XPS).

## Methods

**Figure 1:**
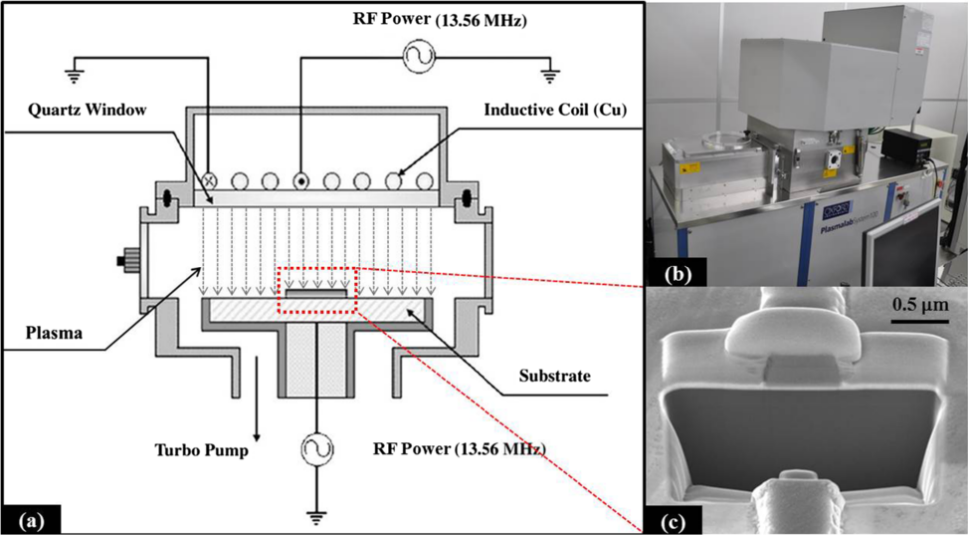
**Schematic diagram and entity of ICP system (a, b) and cross-sectional view of SF**_**6**_**-based etched BaTiO**_**3 **_**film obtained by FIB (c).**

The BaTiO_3_ thin films were deposited by the aerosol deposition (AD) process [15]. The source material for deposition was commercial BaTiO_3_ powder with a particle size of 300 nm. The total thickness of the deposited BaTiO_3_ thin film was approximately 300 nm, which starts from Pt/Ti/SiO_2_/silicon substrate. A Ti (10 nm)/Cr (790 nm) metal shadow mask fabricated by e-beam evaporation is used for the BaTiO_3_ thin films etching. The dry etching process was performed in an ICP system as shown in Figure 1a,b. The etching properties of BaTiO_3_ thin films were investigated in CF_4_/O_2_, C_4_F_8_/O_2_ and SF_6_/O_2_ mixture gas, respectively. The ratios of the three mixture gas between fluorine-based gas and O_2_ are all fixed to 50:5 sccm. The base conditions of the RF power, ICP power, gas pressure and chamber temperature were 150 W, 1,000 W, 7.5 mTorr and 293 K, respectively. The etch rates were measured using focused ion beam (FIB) in order to ensure the high accuracy of measurement. Finally, shadow mask was stripped by hydrofluoric acid and Cr etchant using wet etching to measure the etch rate of the BaTiO_3_ thin film after etching process. The surface morphology of BaTiO_3_ thin film was characterized using AFM. The composition after chemical reaction on the surface of BaTiO_3_ thin film was investigated using XPS. The Al Kα source provides non-monochromatic X-rays at 1,486.6 eV. The survey spectra are taken at a base pressure of 1.1 × 10^−7^ Pa, and a binding energy scan range from 0 to 1,000 eV is sufficient to identify all of the detectable elements. Narrow-scan spectra of all regions of interest are recorded with 23.5 eV pass energy to quantify the surface composition and identify the chemical binding state. The peak of C *1 s* at 285 eV is assigned to carbon from hydrocarbon contamination, and it is used as the criterion to correct the energy of the spectra. The PHI MultiPakTM software (PHI, Chanhassen, MN, USA) is used to fit the narrow-scan spectra of Ba *3d*, Ti *2p*, O *1 s* and F *1 s* for as-deposited and etched BaTiO_3_ films under Shirley-type background subtraction [16]. All of the BaTiO_3_ thin film samples for analysis were set as 1 × 1 cm^2^. The cross-sectional view of the patterned BaTiO_3_ thin film measured by FIB is shown in Figure 1c.

## Results and discussion

### Etching rate and surface morphology

Before analysing the etching rate of the BaTiO_3_ thin films using fluorine-based plasmas, the basic etching behaviour characterizations have to be presented firstly. Actually, the mechanism of the ICP process uses both chemical reaction and physical sputtering. In the CF_4_/O_2_ and C_4_F_8_/O_2_ mixing gas experiment, F^−^ ions from the fluorocarbon (CF_4_ or C_4_F_8_) has strong chemical reactivity. It reacts with BaTiO_3_ thin film to form the low volatile reaction byproducts which include BaF_*x*_ and C_*x*_F_*y*_. Because of the charging effect, these byproducts are adhered to the etched surface. Meanwhile, the various detached CF_*m*_^+^ ions originating from plasma sputter the reaction product from the surface and keep fluoride free to make further chemical reaction [17]. Under the SF_6_/O_2_ plasma environment, F^−^ ions from sulphur fluoride react with BaTiO_3_ thin film. The reacted byproducts such as BaF_*x*_ passivate the surface. In this case, SF_*n*_^+^ ions sputter the reaction product to stimulate the chemical reaction. During etching process, lots of volatile carbonmonoxide, carbondioxide and gaseous sulphur were pumped off by vacuum pumps. In this research, the introduced O_2_ played a role of catalyst, which can enhance the etch rate effectively.

**Figure 2:**
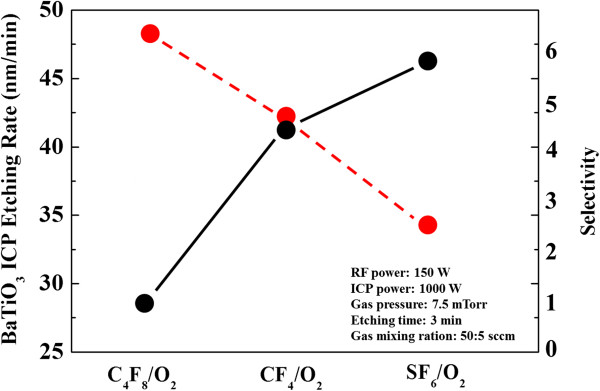
**Etch rates and selectivity of BaTiO**_**3 **_**films.** Etch rates of BaTiO_3_ films (black solid line) and selectivity of BaTiO_3_ to Ti/Cr shadow mask (red dashed line) as a function of the different fluorine-based mixture gas.

The etch rate of the BaTiO_3_ thin film and the etch selectivity of BaTiO_3_ over Ti/Cr metal shadow mask as a function of three different types of mixing of plasmas are shown in Figure 2. Data show that the maximum etch rate is about 46.7 nm/min in SF_6_/O_2_ plasmas. The selectivity achieved was 2.53. As changing the CF_4_ and C_4_F_8_, the etch rate of BaTiO_3_ thin film decreases, which has an etch rate of 41.8 and 27.0 nm/min, while the selectivity achieved was 4.4 and 6.25. Based on the above experimental result, it is disclosed that higher BaTiO_3_ thin film etch rates can be achieved with SF_6_/O_2_ mixture gas compared with CF_4_/O_2_ and C_4_F_8_/O_2_ mixture gas and C_4_F_8_/O_2_ mixture gas is the worst one for BaTiO_3_ thin film etching. Analysing the reasons of the abovementioned result, it is possible to consider for the two following explanations. The first is a minimum amount of ion energy is necessary for SF_6_. The SF_6_-based plasmas can get higher kinetic energy in same condition with CF_4_-based and C_4_F_8_-based plasmas, which accelerate the chemical reactions as well as physical ion bombardment [18]. The second is the lower volatility of fluorocarbon polymers impede the further etching process in C_4_F_8_-based environment as proved by subsequent XPS experiment.

**Figure 3:**
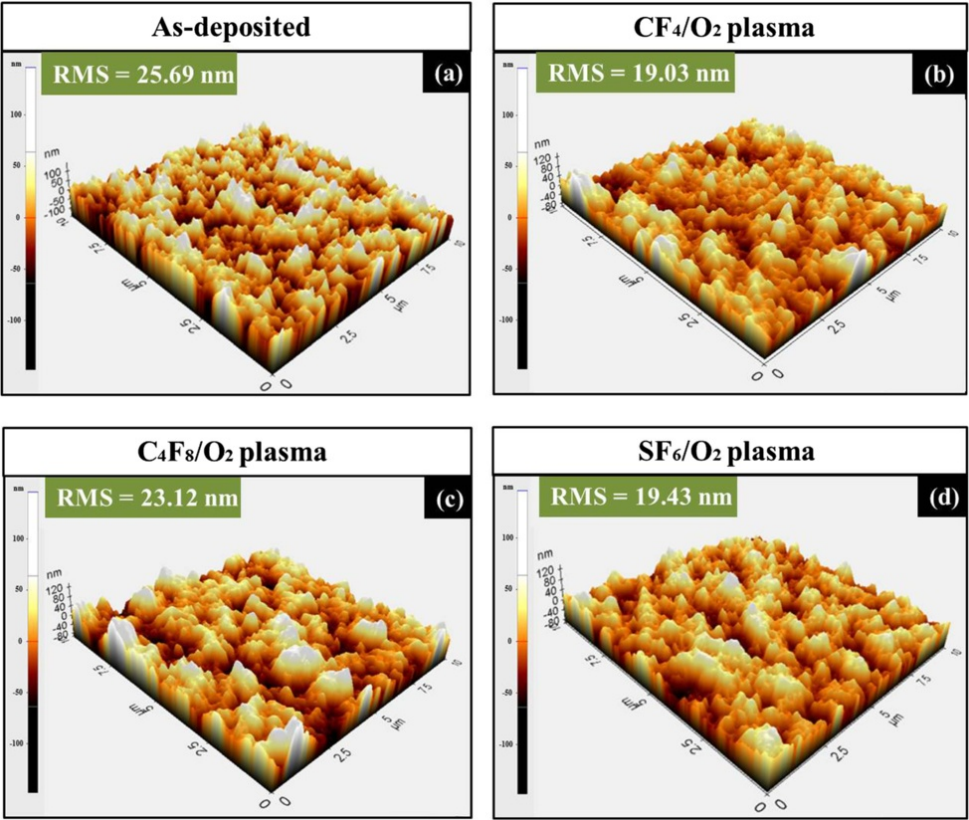
**3-D view of surface morphologies of the BaTiO**_**3 **_**thin films by AFM. (a)** As-deposited, **(b)** etched in the CF_4_/O_2_ plasma, **(c)** etched in the C_4_F_8_ plasma, and **(d)** etched in the SF_6_ plasma. The etching time is set to 3 min, and the scanned areas are all 10 × 10 μm^2^.

**Figure 4:**
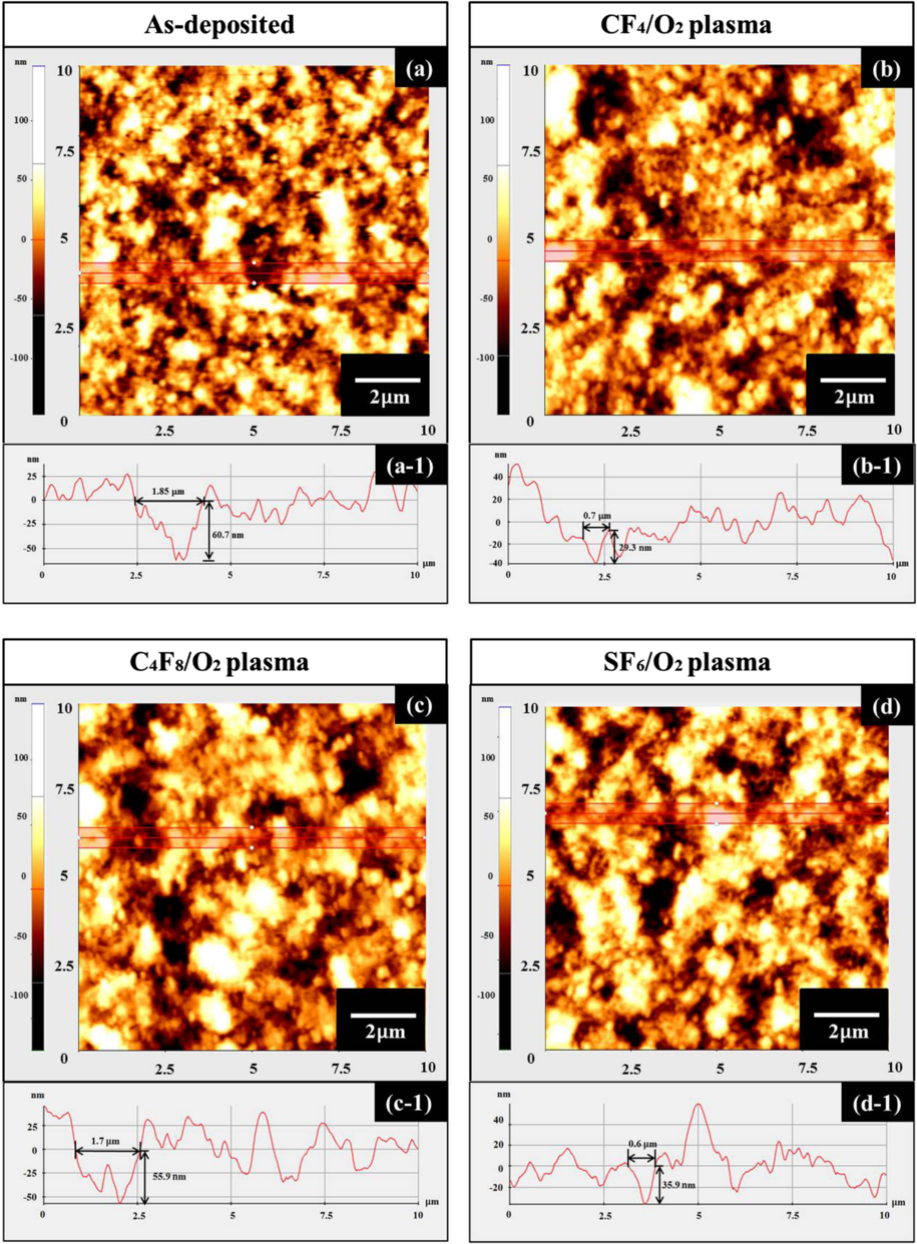
**The surface morphologies of the BaTiO**_**3 **_**films, which are under the unetched and etched by each fluorine-based plasmas. (a, b, c, d)** The AFM top views of the selected areas and (a-1 to d-1) the corresponding cross-sectional surface line profiles of BaTiO_3_ thin films under different conditions.

Figure 3 demonstrates the surface morphologies of the same BaTiO_3_ films which are under the unetched and etched by each fluorine-based plasmas. In each sample, the surface morphologies are investigated by root-mean-square (RMS) roughness and cross-sectional surface line profiles. Figure 3a,b,c,d shows 10 × 10 μm^2^ AFM images of 3-D views. It can be seen that the RMS roughness value of the as-deposited BaTiO_3_ film is 25.69 nm. After the BaTiO_3_ films etched in three fluorine-based plasmas, a better surface morphology can be achieved compared to the unetched examined sample, while there is no obvious difference that appears between the CF_4_/O_2_ etched BaTiO_3_ film surface and SF_6_/O_2_ etched surface. They have a RMS roughness value of 19.03 and 19.43 nm, respectively. However, in the case of C_4_F_8_/O_2_ etched BaTiO_3_ film surface, surface morphology is worse than two others with a RMS roughness value of 23.12 nm. This may attribute to the re-deposition and growth of C_*x*_F_*y*_ polymer during the C_4_F_8_/O_2_ ICP etch [19]. Obviously, the quality of surface morphology of BaTiO_3_ film is deteriorated after etching in C_4_F_8_/O_2_ in comparison with those etched by CF_4_/O_2_ and SF_6_/O_2_. Figure 4a,b,c,d shows the AFM top 2-D views of the selected areas, and the cross-sectional surface line profiles are shown in Figure 4 (a-1 to d-1) corresponding to Figure 4a,b,c,d). The cross-sectional surface line profiles indicate the change in both the diameter and depth of the craters on the surface, which follow the trend in Figure 3a,b,c,d). Figure 4 (a-1) shows the craters on the as-deposited BaTiO_3_ films, which have a diameter of 1.85 μm and a depth of 60.7 nm. After three different fluorine-based plasma etching treatment, the smaller craters can be observed. Two relative high-quality BaTiO_3_ films can be found in CF_4_/O_2_ and SF_6_/O_2_ plasmas as shown in Figure 4 (b-1) and (d-1), which have diameters of 0.7 and 0.6 μm and depths of 29.3 and 35.9 nm, respectively. However, Figure 4 (c-1) reveals that a relative larger craters with a diameter of 1.7 μm and a depth of 55.9 nm appeared on the surface of the thin film.

### XPS analysis

**Figure 5:**
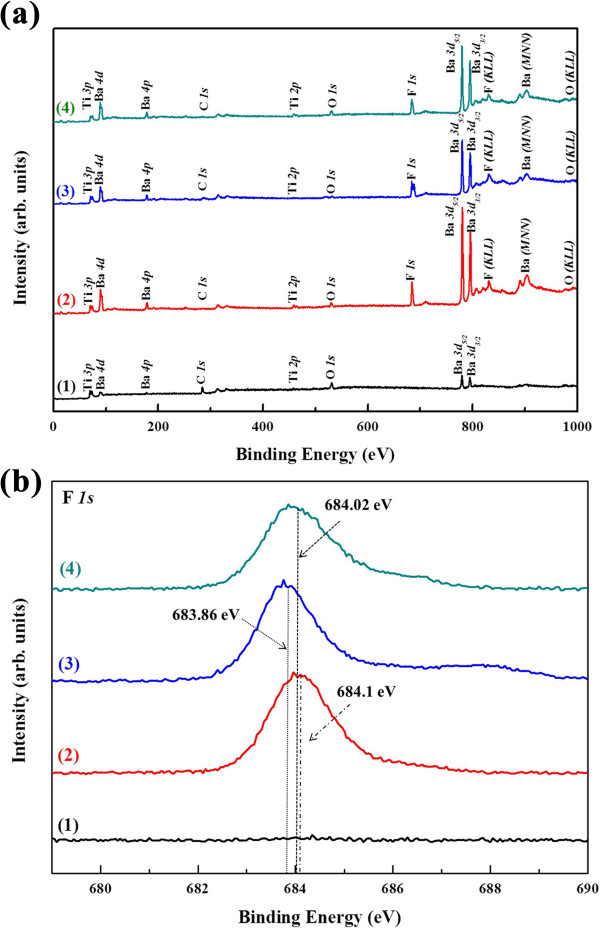
**XPS survey spectrum of BaTiO**_**3 **_**thin films and F *1 s *narrow-scan spectrum for each BaTiO**_**3 **_**thin films. (a)** XPS survey spectrum of BaTiO_3_ thin films: (1) as-deposited, (2) etched in the CF_4_/O_2_ plasma, (3) etched in the C_4_F_8_/O_2_ plasma, and (4) etched in the SF_6_/O_2_ plasma. **(b)** F *1 s* narrow-scan spectrum for each BaTiO_3_ thin films. The vertical dashed line indicates each maximal position of the F *1 s* singlet.

In order to know the more detailed surface chemical composition, an XPS analysis was performed. The XPS survey spectra obtained among the as-deposited and etched BaTiO_3_ films by three different mixture gas are shown in Figure 5a. In Figure 5a (1), the photoelectron lines of Ba, Ti, O and C elements exist on the as-deposited BaTiO_3_ films surface. C *1 s* is used for the criterion to rectify the energy of spectra that has a peak at 285.0 eV from contaminated hydrocarbon [20]. There are Ba, Ti, O, C and F XPS photoelectron lines, where Ba *4d* (89.7 eV) (CF_4_/O_2_ etched), Ba *4p* (178.8 eV) (CF_4_/O_2_ etched), Ba *3d*_*5/2*_ (780.16 eV) (CF_4_/O_2_ etched), Ba *3d*_*3/2*_ (795.75 eV) (CF_4_/O_2_ etched), Ti *3p* (73.5 eV) (CF_4_/O_2_ etched), Ti *2p* (458.1 eV) (CF_4_/O_2_ etched), C *1 s* (285.0 eV) (CF_4_/O_2_ etched), O *1 s* (529.5 eV) (CF_4_/O_2_ etched) and F *1 s* (684.1 eV) (CF_4_/O_2_ etched); Ba *4d* (89.4 eV) (C_4_F_8_/O_2_ etched), Ba *4p* (178.2 eV) (C_4_F_8_/O_2_ etched), Ba *3d*_*5/2*_ (780.55 eV) (C_4_F_8_/O_2_ etched), Ba *3d*_*3/2*_ (795.8 eV) (C_4_F_8_/O_2_ etched), Ti *3p* (74.1 eV) (C_4_F_8_/O_2_ etched), Ti *2p* (459.1 eV) (C_4_F_8_/O_2_ etched), C *1 s* (285.0 eV) (C_4_F_8_/O_2_ etched), O *1 s* (531.4 eV) (C_4_F_8_/O_2_ etched) and F *1 s* (683.8 eV) (C_4_F_8_/O_2_ etched); Ba *4d* (89.4 eV) (SF_6_/O_2_ etched), Ba *4p* (178.7 eV) (SF_6_/O_2_ etched), Ba *3d*_*5/2*_ (779.1 eV) (SF_6_/O_2_ etched), Ba *3d*_*3/2*_ (795.35 eV) (SF_6_/O_2_ etched), Ti *3p* (73.2 eV) (SF_6_/O_2_ etched), Ti *2p* (458.0 eV) (SF_6_/O_2_ etched), C *1 s* (285.0 eV) (SF_6_/O_2_ etched), O *1 s* (529.85 eV) (SF_6_/O_2_ etched) and F *1 s* (684.1 eV) (SF_6_/O_2_ etched) and the valence-type Auger lines for F (KLL) (838.2 eV), Ba (MNN) (902.7 eV) and O (KLL) (990.3 eV) can be confirmed on the three etched BaTiO_3_ film surfaces in Figure 5a (2, 3, 4). Figure 5b shows the XPS narrow-scan spectra of F *1 s* obtained from the BaTiO_3_ films surface in as-deposited and etched by different mixture gas. There is no photoelectron line of the element F in the as-deposited BaTiO_3_ films specimen. After etching in CF_4_/O_2_, C_4_F_8_/O_2_ and SF_6_/O_2_ mixing gas environment, each F *1 s* XPS spectrum shows a wide peak in the region of 682 to 686 eV with a maximum corresponding to a binding energy of 684.1, 683.86 and 684.02 eV, respectively. The fact that the XPS survey spectra of BaTiO_3_ films in Figure 5a is higher consistent with the F *1 s* narrow-scan spectra shown in Figure 5b indicates that chemical reaction occurred when the fluorine-based plasmas were applied into etching process.

**Figure 6:**
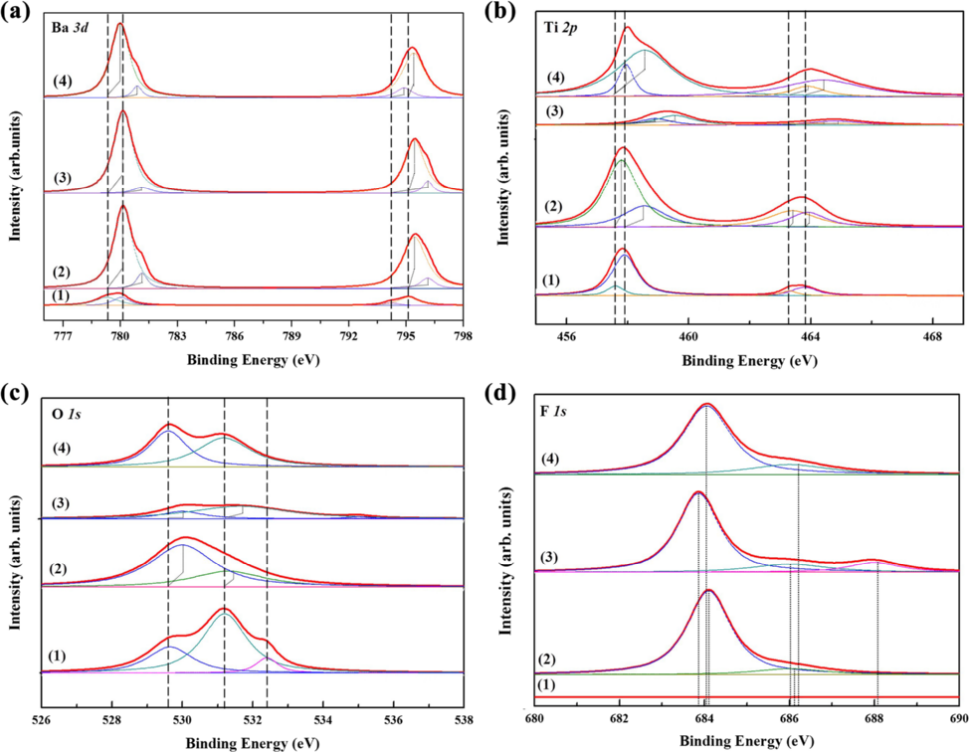
**The narrow-scan spectra of (a) Ba *3d*, (b) Ti *2p*, (c) O *1 s *and (d) F *1 s *peaks for each BaTiO**_**3 **_**film.** (1) As-deposited, (2) etched in the CF_4_/O_2_ plasma, (3) etched in the C_4_F_8_/O_2_ plasma, and (4) etched in the SF_6_/O_2_ plasma. Red bold solid lines represent the fitted experimental results after subtracting Shirley-type background. Peaks with dashed line are fitted sub-peaks. Peak positions of the Ba *3d*, Ti *2p*, O *1 s* and F *1 s* are the average peak positions for the corresponding sub-peaks. The broken lines reveal the approximate chemical shifts.

Figure 6 shows the peaks of the XPS narrow-scan spectra of (a) Ba *3d*, (b) Ti *2p*, (c) O *1 s* and (d) F *1 s*, which were obtained from the BaTiO_3_ film in as-deposited and each different fluorine-based plasma etched environment. Figure 6a shows the photoelectron peaks of Ba *3d*. It can be seen that the unetched doublet consists of two peaks which are observed at 779.9 and 795.1 eV, which are mainly identified as signals from Ba-O bonds. The deconvoluted sub-peaks of Ba *3d*_*5/2*_ and Ba *3d*_*3/2*_ are related to BaCO_3_[21] or a relaxed Ba phase because of the O vacancies and the cation defects [22]. After the BaTiO_3_ thin films were exposed to the CF_4_/O_2_ and C_4_F_8_/O_2_ plasma severally, the peaks of Ba *3d*_*5/2*_ and Ba *3d*_*3/2*_ were chemically shifted to a higher binding energy and the maximum deviation are about 0.26/0.255 and 0.65/0.70 eV in comparison with the unetched counterparts. After the treatment in SF_6_/O_2_ plasma, Ba *3d*_*5/2*_ and Ba *3d*_*3/2*_ peaks show higher binding energy shifts of 0.1 and 0.25 eV, respectively. The shift of peaks indicates that Ba chemically reacted with F-component species, which some Ba-O bonds are broken and a few Ba-F bonds are generated. Because the bonding energies of the Ba-F bonds are higher than Ba-O bonds [23], peaks of the BaTiO_3_ film shift towards higher binding energy.

Figure 6b shows the photoelectron peaks of Ti *2p* from the as-deposited and etched BaTiO_3_ films surface. In Figure 6b (1), the unetched Ti *2p* consists of two wide peaks of Ti *2p*_*3/2*_ (457.8 eV) and Ti *2p*_*1/2*_ (463.57 eV) due to Ti-O bonds. After etching in CF_4_/O_2_, C_4_F_8_/O_2_ and SF_6_/O_2_ plasma, the peaks of Ti *2p*_*3/2*_ and Ti *2p*_*1/2*_ shift towards higher binding energy regions by 0.05 and 0.23, 1.35 and 0.98, and 0.2 and 0.43 eV, respectively, which is shown in Figure 6b (2, 3, 4). When BaTiO_3_ film is etched in C_4_F_8_/O_2_ plasma, the intensity of the Ti *2p*_*3/2*_ and Ti *2p*_*1/2*_ peaks decreased obviously because of the higher volatility of byproduct TiF_*x*_. The byproduct TiF_*x*_ can be partly removed from the film surface as the thermal desorption process. The reason why the chemical shifts towards higher binding energy can be explained by the theory of bond shift compensation scheme between TiF_*x*_ and the etched BaTiO_3_ film [24].

The fitted O *1 s* narrow scan spectra of each BaTiO_3_ sample is shown Figure 6c. An O *1 s* (531.24 eV) peak of the as-deposited BaTiO_3_ film which consists of three sub-peaks located at 529.65, 531.2 and 532.4 eV is shown in Figure 6c (1). The three sub-peaks are mainly affected by Ba-(O *1 s*) (780 eV), Ti-(O *1 s*) (529 eV) and C-(O *1 s*) (532.3 eV) bonds [20]. The two oxides of Ba are made up of BaO and TiO_2_ in the BaTiO_3_ film, the surface contamination introduced the C-O bonds. The shoulder located at 532.4 eV is ascribed to the surface water vapour and carbon dioxide. In this research, the BaTiO_3_ film was deposited by AD method, the surface phase was formed with water vapour and carbon dioxide inevitably. After etching in each fluorine-based plasma, the etched film shows a chemical shift towards higher binding energy region, which is demonstrated in Figure 6c. It is revealed that the disconnection between Ba-O and Ti-O and re-connection between Ba-F and Ti-F happened through the physical sputtering of CF_*m*_^+^ and SF_*n*_^+^ ions and chemical reactions with reactive fluorides. A phenomenon can be observed that the sub-peaks at 532.4 eV is disappeared after etching in different fluorine-based plasmas. The reason of the decrease of sub-peaks in Figure 6c (2, 3, 4) compared with Figure 6c (1) is that the physical bump of ions removed the surface contamination (carbon dioxide) and the etching process is in the vacuum conditions, which would not introduce secondary contamination. Therefore, the sub-peaks at 532.4 eV in Figure 6c (2, 3, 4) cannot be found anymore.

Figure 6d shows the F *1 s* narrow-scan spectra of the as-deposited and each etched BaTiO_3_ film surface. As shown in Figure 6d (1), there is no signal from a fluorine-contained compound. While adding the etching reaction CF_4_/O_2_ and SF_6_/O_2_ plasma for each sample, F *1 s* appear at the binding energy of 684.1 and 684.02 eV, as revealed in Figure 6d (2 and 4). The sub-peaks are situated at 684.1/686.1 and 684.02/686.2 eV, respectively, which are assigned to the product of the etching reaction of Ba-F and a residue of Ti-F [25]. After etching in C_4_F_8_/O_2_ plasma, the F *1 s* signal emerged and consisted of three sub-peaks (683.86, 686.01 and 688.15 eV). Unlike the CF_4_/O_2_ and SF_6_/O_2_ plasma, the main contributions of these three sub-peaks result from Ba-F, Ti-F and a residue of C-F compounds [26].

## Conclusions

In this present work, an investigation of dry etching mechanisms for BaTiO_3_ thin films in ICP system using different fluorine-based plasmas was carried out. Experimental results indicate that a higher BaTiO_3_ thin film etch rates were achieved with SF_6_/O_2_ plasmas. The etch rate of SF_6_/O_2_ plasmas is over than 46.7 nm/min at RF power/ICP power of 150/1,000 W under gas pressure of 7.5 mTorr. The result of AFM reveals that the roughness of all etched surfaces by fluorine-based plasmas ameliorated in comparison with the as-deposited surface. Moreover, a better etched surface morphology can be achieved using SF_6_/O_2_ plasmas. Chemical compositions and bonding states on as-deposited and each etched BaTiO_3_ thin films were investigated by XPS. The XPS analysis indicated the accumulation of reaction products. According to the comprehensive analysis and comparison, SF_6_-based plasmas showed higher etch rates and excellent surface morphology. In addition, in terms of recent severe environment, SF_6_ gas is not a potent greenhouse gas compared with other two greenhouse effect gas CF_4_ and C_4_F_8_. SF_6_-based plasmas can be recommended to be an ideal candidate gas for BaTiO_3_ dry etching.

## Competing interests

The authors declare that they have no competing interests.

## Authors’ contributions

YL conceived of the study, managed the entire study and drafted the manuscript. CW, ZY and HKK performed the fabrication and the measurements. As the corresponding author, NYK provided the overall research conception, guided the research and revised the manuscript. All authors read and approved the final manuscript.
